# Nutritional Components and Bioactive Substances of Colored Rice: From Molecular Formation, Nutritional and Health Benefits to Industrial Application Prospects

**DOI:** 10.3390/molecules31122149

**Published:** 2026-06-18

**Authors:** Donghong Lai, Yuehong Peng, Han Wu, Qiangqiang Xiong

**Affiliations:** 1 Jiangsu Key Laboratory of Crop Genetics and Physiology/Jiangsu Key Laboratory of Crop Cultivation and Physiology, Agricultural College of Yangzhou University, Yangzhou 225009, China; 17770128045@163.com (D.L.); 17680494958@163.com (Y.P.); 18118275573@163.com (H.W.); 2 Jiangsu Co-Innovation Center for Modern Production Technology of Grain Crops, Yangzhou University, Yangzhou 225009, China

**Keywords:** colored rice, anthocyanins, nutritional quality, chronic disease, molecular breeding

## Abstract

Colored rice is a type of functional cereal rich in bioactive substances such as anthocyanins. This article systematically reviews its molecular formation, nutritional quality, health effects, and industrial applications. At the molecular level, the biosynthesis of pigments such as anthocyanins is regulated by transcription factors including MYB and bHLH, and is influenced by environmental conditions such as light, temperature, and fertilization. Nutritional analysis shows that, compared to white rice, colored rice contains higher levels of resistant starch, high-quality protein, dietary fiber, minerals, and vitamins. In addition, it is rich in various phenolic compounds and gamma-aminobutyric acid (GABA). These bioactive components have functional food applications in chronic diseases such as diabetes, cardiovascular diseases, and cancer through multiple mechanisms. These mechanisms include antioxidant and anti-inflammatory activities, regulation of glucose and lipid metabolism, and modulation of the gut microbiota. Despite the advancements in molecular breeding and precision cultivation technologies that have driven variety improvement and diversified product development, the industry still faces challenges such as the contradiction between nutrient retention and processing palatability, as well as insufficient market recognition. In the future, it is necessary to integrate multidisciplinary technologies to promote the development of colored rice. This may contribute to modulating risk factors associated with chronic diseases based on precision nutrition evidence.

## 1. Definition, Classification, and Resource Distribution of Colored Rice

**Figure 1:**
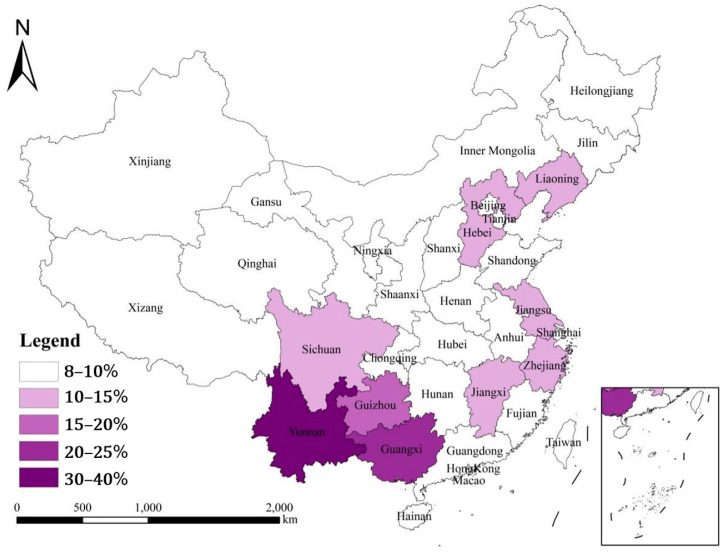
Distribution map of colored rice in China.

Rice (*Oryza sativa* L.) is one of the most important food crops in the world. China, as one of the earliest origins of rice, boasts abundant germplasm resources. Rice is distributed throughout the country, mainly in the vast areas south of the Yangtze River basin. Conventional rice is most commonly white rice, while colored rice refers to an important specific rice germplasm resource formed by the deposition of different pigments in the seed coat or pericarp, that is, the caryopsis (brown rice) exhibits different colors, which can be subdivided into black, red, yellow, green, etc., with black (purple) rice being the most common [1,2]. Colored rice varieties originate from wild rice and are distributed in regions of China with abundant rice germplasm resources and good ecological diversity. China possesses 90% of the world’s colored rice variety resources, while the remaining 10% of colored rice varieties are mainly distributed in Southeast Asian countries such as India, Bangladesh, Indonesia, Japan, Vietnam, and the Philippines [3,4]. Currently, among the rice germplasm resources preserved in China, colored rice varieties account for about 10%, of which 8963 are red rice varieties, ranking first among colored rice varieties [5]. Black rice is widely cultivated in the southwest region of China (Yunnan, Sichuan, Guizhou, etc.), the northeast region (Jilin, Liaoning, etc.), as well as in Guangxi, Hunan, Jiangsu, and other places (Figure 1). For example, local varieties such as “Dianxiangzi No. 1” in Yunnan, “Donglan Momi” in Guangxi, “Yaxue Nuo” in Changshu, and “Guiyu Hei Nuo” in Guangxi have attracted much attention due to their high anthocyanin content and strong stability [6,7,8]. During the long-term natural evolution process, wild rice has undergone some color variations in response to environmental pressures (such as pest resistance) [9]. These variations are the result of biological evolution and the natural basis for the emergence of colored rice. However, in the process of human cultivation and domestication, the genes controlling anthocyanin synthesis carried by colored rice are closely linked to genes controlling seed shattering and dormancy, resulting in colored varieties that are prone to shattering and have low germination rates during cultivation [1]. Therefore, after long-term artificial and natural selection, most colored rice varieties have gradually been eliminated. Later, with the efforts of breeders, some excellent colored rice varieties were selected and cultivated, and then applied in production. With the increase in market demand and the deepening of related research, Chinese breeders have gradually cultivated many distinctive local colored rice varieties, providing conditions for basic research on rice genetics, genomics, and variety improvement.

## 2. Molecular Formation and Accumulation Mechanism of Characteristic Components in Colored Rice

### 2.1. Chemical Structure and Types of Pigment Substances in Colored Rice

**Figure 2:**
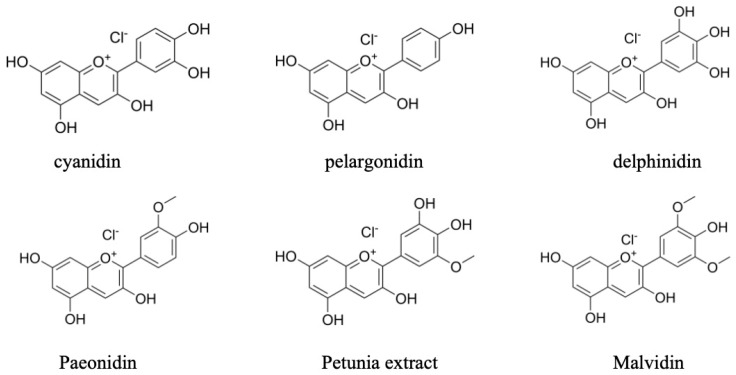
Chemical structural formulas of six common anthocyanins in colored rice.

Anthocyanins, along with beet pigments, carotenoids, and chlorophyll, are collectively known as the four major types of plant pigments. They are flavonoid compounds containing multiple phenolic hydroxyl groups, widely distributed in plant roots, stems, leaves, flowers, and fruit tissues, giving plants vibrant colors such as blue, purple, and red. The pigment substances in colored rice mainly include anthocyanins, carotenoids, flavonoid compounds, etc. Among them, anthocyanins are the key components determining the color of rice. There are more than 20 types of anthocyanins known in nature [10]. Colored rice mainly contains the following six common anthocyanins: cyanidin, which is the dominant pigment in black rice (accounting for about 75%). Pelargonidin, a deep red pigment, is the main pigment in red rice. Delphinidin, a blue-purple pigment, is present in some black rice varieties, as well as glycoside derivatives such as peonidin, petunidin, and malvidin (Figure 2) [11,12]. Chlorophyll is primarily found in green rice, imparting its green color and possessing antioxidant properties; carotenoids, as accessory pigments in photosynthesis, are present in various colored rice varieties, offering stress resistance and nutritional value. The stability of chlorophyll in green rice during processing and storage is significantly lower than that of anthocyanins in black rice and red rice. Chlorophyll is highly sensitive to heat, light, pH changes and enzymatic hydrolysis. It is prone to de-methylation reactions and degradation during conventional heat processing or long-term storage, resulting in the loss of green color and transformation into olive-brown. Anthocyanins are relatively stable under acidic conditions. The multiple phenolic hydroxyl groups in their molecular structure give them stronger antioxidant properties, enabling them to maintain better color retention under appropriate storage conditions. These pigments not only affect the appearance of rice but also exhibit biological activities such as antioxidation and anti-inflammation. For instance, the free radical scavenging ability of anthocyanins is significantly higher than that of vitamin E and vitamin C, which contributes to delaying aging and improving metabolic health [13]. It has potential value in enhancing the nutritional value and health functions of colored rice.

### 2.2. Molecular Pathways and Regulatory Mechanisms of Pigment Biosynthesis

**Figure 3:**
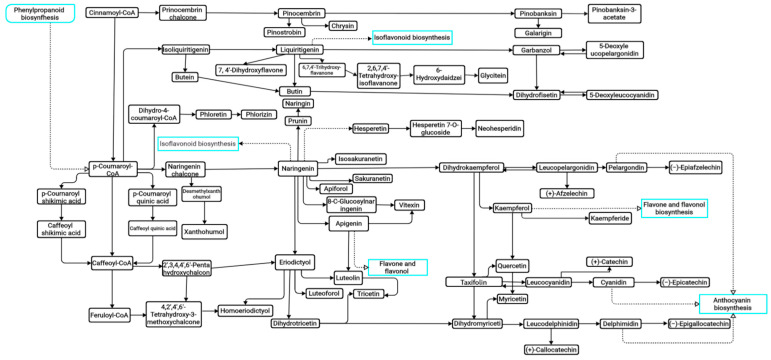
Flavonoid biosynthetic pathway. The blue box represents the KEGG metabolic pathway.

The biosynthesis of colored rice germplasm pigments mainly involves the flavonoid pathway’s anthocyanin and proanthocyanidin metabolic branches (Figure 3). The initial substance of this pathway is phenylalanine, which is gradually transformed into anthocyanins and proanthocyanidins through the phenylpropanoid pathway and a series of enzymatic reactions. The regulatory mechanism of colored rice pigment biosynthesis involves multiple levels, including gene regulation (structural genes, regulatory genes, gene interactions) and the synergistic effect of environmental factors. In the gene regulation network, the three types of transcription factors, *MYB*, *bHLH*, and *WD40*, are the core elements regulating anthocyanin synthesis. These three often form a highly conserved *MBW (MYB-bHLH-WD40)* transcriptional complex, which collaboratively activates the expression of downstream structural genes [9]. Specifically, the *WD40* transcription factor serves as the backbone protein of the *MBW* complex, mainly mediating protein–protein interactions through its *WD40* repeat domain, providing a stable binding platform for *MYB* and *bHLH*, thereby enhancing the transcriptional activation activity of the entire complex. Although *WD40* proteins usually do not directly bind to *DNA*, they play an indispensable role in stabilizing the complex conformation, promoting nuclear localization, and regulating the activity of the complex. Therefore, the synergy of *MYB*, *bHLH*, and *WD40* is indispensable and together constitutes the core transcriptional regulatory network for regulating the pigment synthesis of colored rice. In addition, regulatory genes can regulate the expression of structural genes in the anthocyanin synthesis pathway either individually or jointly (Table 1), thereby controlling the accumulation of anthocyanins in the plant [14,15]. Anthocyanins are primarily accumulated in the fruit epidermis, which is greatly influenced by light exposure. For example, the synthesis of anthocyanins in purple tomato fruits is induced by light. Purple tomato (No. 19861) is a type of tomato containing anthocyanins. After the ovary expands, anthocyanins begin to accumulate in the areas of the epidermis exposed to sunlight, gradually deepening the purple color [16]. Insufficient light can lead to strawberries in the market having a pale color and an unsweet taste. Light signals have an impact on the regulatory mechanism of strawberry anthocyanin biosynthesis [17]. Biofortification is a strategy aimed at increasing the levels of micronutrients or phytochemicals in crops through conventional breeding or genetic engineering methods. For example, Golden Rice has been genetically engineered to produce β-carotene [18]. Astaxanthin is another important compound, with antioxidant activity stronger than other carotenoids or vitamin E. Recent studies have explored using transgenic approaches to elucidate the extended biosynthetic pathways of carotenoids in plants, including ketocarotenoids and astaxanthin. In addition, researchers have identified the transgenes required for endosperm-specific synthesis of lutein in rice. By utilizing high-efficiency multi-gene vector systems such as TGSII and efficient transgene stacking technologies, researchers have developed the world’s first novel functional nutritional rice germplasm with endosperm rich in astaxanthin, known as ‘Astaxanthin Rice’ [19]. By creating a new type of “ruby rice” through dual-gene co-expression, the biosynthesis of betacyanin is achieved in the rice endosperm, significantly increasing the betacyanin content in the endosperm. The antioxidant activity of the rice grain extract is 1.36 to 6.44 times higher than that of the wild type [20]. Some studies have achieved the production of functional rice rich in melatonin through new metabolic engineering strategies, elucidating the molecular mechanism of the mutual regulation between abscisic acid (ABA)-induced serotonin. ABI5 directly binds to key genes (*TDC1*, *TDC3*, *T5H*) in the serotonin synthesis pathway, significantly enhancing the activity of these genes. The activation of these key genes leads to a significant increase in the content of serotonin and melatonin in rice [21]. Currently, research on the anthocyanin synthesis pathway is relatively mature, with some important structural and regulatory genes being cloned in different species, and research on related key genes is also relatively in-depth. However, the regulatory mechanism and function of certain regulatory genes in the metabolic pathway are still unclear [14].

### 2.3. The Deposition and Distribution Pattern of Pigments in Grains

Research indicates that anthocyanins, a type of flavonoid compound, are natural water-soluble flavonoid pigments produced through the phenylpropane pathway [22]. Anthocyanins are the largest subclass of plant flavonoids, widely distributed in various plant tissues, providing bright colors for flowers, fruits, vegetables, and grains [23]. In colored rice, anthocyanins are mainly deposited in the seed coat and aleurone layer, while carotenoids are primarily distributed in the embryo and endosperm. The accumulation of these pigments undergoes dynamic changes during grain development. At the early stage of development, the caryopsis of colored rice appears green. Four to six days after pollination, anthocyanins begin to deposit in the region of the ovary wall connected to the caryopsis and style. By seven to nine days after pollination, the deposition range of anthocyanins gradually expands, mainly accumulating on the side of the caryopsis near the palea. When pollination enters the 10th to 12th day, anthocyanins significantly increase in the embryo end, distal end, and dorsal vascular bundles of the caryopsis pericarp. From the 13th to the 18th day after pollination, as anthocyanins continue to accumulate, the caryopsis is colored overall, and the color depth continuously increases [2,24]. The degree and pattern of seed coat coloring vary among different varieties. The coloring of the grains is also related to the ratio of anthocyanins to proanthocyanidins. The higher the proportion of anthocyanins, the darker the caryopsis; the higher the proportion of proanthocyanidins, the redder the caryopsis. Green rice, as a rare variety among colored rice, exhibits a unique green color due to the deposition of chlorophyll in its seed coat, while the rice husk appears purple or red [5,14]. Studies have determined that the deeper the color of the seed coat of colored rice, the higher the content of anthocyanins, flavonoids, and other compounds in the rice grains, leading to a stronger overall antioxidant capacity [25]. Research has also found that 7, 14, 21, 28, and 35 days after the grain filling of purple rice, as the grain filling time progresses, the color deepening is positively correlated with the accumulation of minerals, anthocyanins, and flavonoids, and the higher the content of these substances, the stronger the total antioxidant capacity of the grains [26,27].

### 2.4. Influence of Environmental and Genetic Factors on Pigment Accumulation

The deposition and distribution patterns of pigment in the seed coat of colored rice are influenced not only by pigment gene expression and gene interactions but also by environmental and genetic factors. Environmental factors are primarily influenced by light, temperature, moisture, soil, fertilizer, and exogenous spraying [28]. For instance, shading at different growth stages and varying shading intensities, as well as different temperatures, have an impact on the yield, anthocyanin content, and antioxidant capacity of colored rice [29,30]. Studies have found that in tropical regions, a recommended fertilization scheme of 30% organic fertilizer combined with 70% inorganic fertilizer not only synergistically enhances the yield, nitrogen utilization efficiency, and grain anthocyanin content of the current season’s colored rice, but also improves soil quality and significantly increases the yield of subsequent rice crops [31]. Furthermore, different nitrogen application rates affect the pigment content of black rice and red rice. With increasing nitrogen application rates, the secondary metabolites content of Yunnan black rice and red rice initially increases and then decreases, reaching a maximum during the mid-to-late grain filling stage (24–30 days after flowering) and then declining during the mature stage (36 days after flowering) [32]. Other studies have found that exogenous spraying of Se, Zn, Fe, and P elements on the leaves of colored rice can significantly increase the mineral trace element content and bioavailability of colored rice grains [33,34]. Spraying Fe on the leaves of colored rice promotes flavonoid metabolism, enhances antioxidant capacity, and affects glycosylation of flavonoid compounds [35].

Regarding the genetic mechanism of colored rice grain traits, different scholars have proposed various viewpoints. Some scholars believe that this trait is controlled by a pair of dominant genes; other studies suggest that it may be jointly determined by two pairs of independently inherited complementary dominant genes; there are also views that the inheritance of this trait involves more than two pairs of dominant genes or a polygenic system. For example, some studies have shown that the red pericarp of rice is jointly controlled by the *Rc/rc* and *Rd/rd* gene pairs, while the purple pericarp is determined by the interaction of the Pb and Pa genes [36]. These genes (*Ra*, *Rc*, *Rd*, *OsC1*, *OsC2*, etc.) are key genes involved in the formation of colored rice [37], These genes determine the red, black, purple and other colors of rice grains by affecting the accumulation of anthocyanins and anthocyanins, playing a key role in regulating the color of colored rice, Its skin, seed coat, and aleurone layer also deposit abundant anthocyanins. A study used 58 pairs of SSR primers to analyze the genetic diversity of 41 colored rice resources in Heilongjiang Province and 1 colored indica rice resource in Yunnan Province. It was found that the overall genetic diversity differences in the 41 selected colored rice varieties in Heilongjiang Province were relatively small, and the genetic backgrounds were generally similar, indicating that the genetic basis of colored rice in Heilongjiang Province is relatively narrow [38]. Sedeek and others used CRISPR-Cas9 gene editing technology to successfully knock out three flowering time inhibitor genes (*Hd2*, *Hd4*, and *Hd5*) in Indonesian black rice. Through this method, they improved the long lifecycle and limited productivity of this variety, ultimately obtaining a new early-maturing black rice line with a compact plant type [39]. Subsequent breeding workers can further improve the functional research of the rice genome by linking the pigment genes, drop grain genes, seed germination genes, and other genes of colored rice. Simultaneously editing multiple target genes can significantly accelerate the improvement of agronomic performance in these rice varieties. In addition, fine-tuning the expression of target genes by editing regulatory regions can help balance and improve nutritional characteristics and grain yield.

**Table 1:** The key genes involved in the biosynthesis of pigments such as flavonoids in rice.

Gene Name	Genetic Type/Encoded Product	Main Functions and Purposes	References
*bHLH*	Transcription factor (basic helix-loop-helix protein)	It cooperates with transcription factors such as *MYB* and *WD40* to regulate the expression of structural genes in the anthocyanin synthesis pathway, thereby controlling the accumulation amount of anthocyanins.	[14,15]
*MYB*	Transcription factor (myeloid cell proliferation protein)	As a key regulatory factor, it participates in regulating the biosynthesis pathway of anthocyanins and can form a complex with *bHLH* to activate downstream structural genes.	[14,15]
*WD40*	Transcription factor (*WD40* repeat protein)	Participates in the formation of the *MYB-bHLH-WD40 (MBW)* transcriptional complex and cooperatively regulates the expression of multiple structural genes in the flavonoid pathway.	[14]
*TDC1*, *TDC3*, *T5H*	Genes of key enzymes in the serotonin synthesis pathway	It is directly bound and activated by the transcription factor ABI5, and its upregulated expression can significantly increase the content of serotonin and melatonin (functional components) in rice.	[21]
*Rc*	The gene regulating the color of the seed coat	It jointly controls the formation of the red seed coat in rice and is a key gene for the accumulation of red rice pigment (proanthocyanidins).	[36,37]
*Rd*	The gene regulating the color of the seed coat	It has a complementary effect with the *Rc* gene, determining the red seed coat phenotype and influencing the accumulation of proanthocyanidins.	[36,37]
*Pb*	Purple pericarp regulatory gene	Interacts with the Pa gene, controlling the formation of purple seed coats in rice and influencing the accumulation of anthocyanins.	[36]
*Pa*	Purple pericarp regulatory gene	It jointly determines the purple seed coat trait with the *Pb* gene and participates in the regulation of anthocyanin biosynthesis.	[36]
*Ra*	Genes related to pigment synthesis	It is listed as one of the key genes for the formation of colored rice, influencing the deposition of pigments in the grains.	[37]
*OsC1*	*MYB* family transcription factors	The key *MYB* genes regulating anthocyanin synthesis in rice affect the accumulation of pigments in tissues such as the seed coat and leaves.	[37]
*OsC2*	*MYB* family transcription factors	Similar to the function of *OsC1*, it participates in regulating the anthocyanin synthesis pathway and affects the pigment accumulation pattern.	[37]
*Hd2*, *Hd4*, *Hd5*	Flowering time inhibitory gene	By using CRISPR-Cas9 to knockout these genes, the flowering time and plant height of black rice can be improved, thereby indirectly influencing its agronomic traits and adaptability.	[39]

## 3. Nutritional Components and Physiologically Active Substances of Colored Rice

**Figure 4:**
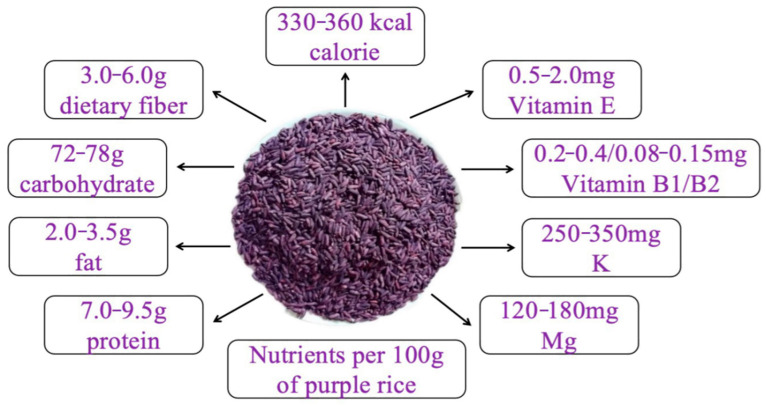
The main nutritional components of purple rice per 100 g.

Rice is rich in carbohydrates, proteins, fats, amino acids, mineral elements, vitamins, and other nutrients (Figure 4) [40], and is an important nutritional source for maintaining human health and development. Studies have shown that insufficient intake can hinder normal body development and induce a series of adverse health problems [41]. Rising living standards have been accompanied by a shift in consumer preferences toward rice varieties that offer greater nutritional and health value. As a staple food, there are significant differences in the nutritional composition of rice among different varieties. Among them, colored rice has higher or lower levels of certain physiologically active substances or special components that are beneficial to human health, such as the skin and embryo, compared to ordinary rice. It has the ability to regulate human physiological metabolism and meet the needs of different consumer groups and special consumer groups, making it a specialized and health-promoting rice [42]. In-depth research on the formation process of nutritional components in rice varieties with different colored grains is of great significance for the development of health products using rice grains as raw materials. Therefore, improving the nutrition, functionality, and safety of rice is a common concern among disciplines such as crop genetics and breeding, food science, and nutritional medicine.

### 3.1. Macronutrients in Colored Rice

Compared with conventional rice, colored rice contains richer macronutrients and micronutrients such as lipids, carbohydrates, proteins, vitamins, and minerals. In particular, it contains physiologically active substances such as vitamin C, carotenoids, flavonoids, cardiac glycosides, and alkaloids, which are lacking in conventional rice. These substances mainly exist in the skin, seed coat, aleurone layer, embryo, and endosperm, and have more comprehensive nutritional components than conventional rice [43,44]. Colored rice contains 75% carbohydrates, mainly starch and a small amount of polysaccharides (resistant starch, cellulose, hemicellulose, and pectin), which provide the energy needed for daily life. Protein is the main component, second only to starch, which affects the edible and nutritional quality of rice [2]. The biological value of rice protein is second only to eggs, and it is rich in essential amino acids for the human body. It contains high-quality first limiting amino acid—lysine, and has low allergenicity, making it a high-quality source of plant protein [45]. The fat characteristics of colored rice are also prominent. Fat exists in the endosperm layer and rice bran layer of colored rice, and is a good source of linoleic acid, oleic acid, palmitic acid, and other essential fatty acids. Moreover, the fat content of colored rice is usually higher than that of regular white rice, but there are certain differences between different varieties. The distribution of rice fat is uneven, with 51% concentrated in the rice embryo, 32% in the surface layer, and only 17% distributed in the endosperm layer. Moreover, the distribution of fat in the endosperm layer is also uneven, with denser starch crystals and lower fat content towards the inner core area. In the process of rice processing, brown rice is ground to remove rice bran (including seed coat, endosperm layer, and embryo) and then made into polished rice. With the continuous improvement of grinding accuracy, the lipid content in rice will decrease accordingly [46]. If the fat content of brown rice is below 4%, the fat content of polished rice is generally below 2%. There are many factors that affect the fat content of rice, and the fat content of different varieties of rice varies greatly. Japonica rice usually has a higher fat content than indica rice [47]. Different cultivation conditions, such as temperature, light [48], and fertilizer application, can also affect [46].

### 3.2. Trace Nutrients in Colored Rice

Vitamins are essential nutrients for humans and represent an important metric for assessing the nutritional quality of rice. B vitamins, as important members of the vitamin family, are water-soluble vitamins essential for maintaining normal bodily functions and metabolic activities. They are essential nutrients that must be obtained from dietary sources due to the body’s inability to synthesize them [49]. Previous studies have measured the vitamin content between different colored rice varieties and found that the VB2 and VE contents of six colored rice varieties were significantly higher than those of white rice, with increases of 9.1% to 134.0% and 29.2% to 109.1%, respectively. Black rice exhibited a significantly higher vitamin B_2_ content than red rice, while white rice showed the lowest levels among the three varieties tested [50]. VB1 and VB2 can regulate hormone levels and shorten hemostasis time, which is of great significance in the adjuvant treatment of functional uterine bleeding [51]. VB2 can reduce the occurrence of Alzheimer’s disease [52]. Studying the content of B vitamins in rice and exploring germplasm resources rich in B vitamins are of great significance for obtaining more vitamin B from rice. Both black and red rice are rich in essential minerals typically deficient in common rice varieties, with black rice showing high iron and zinc levels linked to its anthocyanin content, and red rice providing abundant iron, zinc, and calcium. Scholars have measured 68 rice varieties, including 35 white rice varieties, 17 black rice varieties, and 16 red rice varieties. Compared to white rice, colored rice exhibits superior mineral content, with higher concentrations of calcium, iron, magnesium, manganese, and zinc [53]. Scholars also used the AAS method to determine the content of four mineral elements in 27 colored rice and 34 ordinary rice brown rice planted under the same ecological conditions in Mile County, and compared the content of Fe, Zn, Cu, and Mn in colored rice and ordinary rice. The results showed that the content of four mineral elements in colored rice was significantly higher than that in colorless rice [54]. The International Rice Research Institute has also taken the lead in conducting genetic breeding research on high trace element rice. The most representative achievement is the breeding of an iron-rich and high-yielding rice variety IR164, with an iron content of 25 mg/kg, which is about 60% higher than ordinary rice [42].

### 3.3. Main Physiologically Active Substances in Colored Rice

In recent years, research has found that colored rice is rich in various bioactive compounds that are beneficial to human health, including polyphenols, flavonoids, anthocyanins, various amino acids, phenolic acids, melanin, etc. [55]. These metabolites not only give rice a rich color but also endow it with various nutritional and health functions. Flavonoids belong to the large family of phenolic compounds, also known as polyphenols [53]. Flavonoids, including flavonoids, flavonols, and isoflavones, are commonly found in tea [56] and citrus fruits [57], Studies have shown that consuming three more servings of flavonoid rich foods such as tea and purple rice in our daily lives can reduce women’s risk of three aging related indicators (weakness, decreased physical function, and poor mental health) by 6% to 11%; The risk of male mental health problems has decreased by 15% [58]. Flavonoids play a key role in preventing cardiovascular diseases by reducing cholesterol and triglycerides, inhibiting platelet aggregation, and preventing thrombosis [56]. In addition, colored rice, abundant in phenols and flavonoids, exhibits potent reducing activity and has been found to suppress breast cancer, melanoma, and oral cancer cells. Polyphenols also have the functions of clearing free radicals and antioxidation, which can reduce high-fat and high cholesterol, improve the body’s antioxidant capacity, and reduce oxidative damage to arterial wall cells and other components [59]. By identifying these metabolic markers (such as specific anthocyanin derivatives, glutathione-related metabolites, etc.), it can be determined which substances are directly involved in the antioxidant process. Through biomarker analysis, the biosynthesis pathways of antioxidant substances (such as flavonoid biosynthesis pathway, anthocyanin biosynthesis pathway, etc.) can be traced, revealing the role of key genes in antioxidant formation and providing targets for genetic improvement [60]. As a key measure of nutritional quality in rice, amino acid content—along with the functional role of its metabolites—plays an essential part in rice quality formation [61]. Improving the amino acid content in rice, especially essential amino acids such as lysine and gamma aminobutyric acid, is an important goal for enhancing rice quality. Related studies have found that consuming rice rich in gamma aminobutyric acid has effects on regulating blood pressure, improving liver and kidney function, and enhancing sleep quality in the human body [62]. Black rice has a high content of anthocyanins, with a total anthocyanin mass fraction of 0.3–4.7 mg/g [63]. The main components of black rice anthocyanins include cyanidin-3-glucoside (81.99%), cyanidin-3,5-diglucoside, cyanidin-delphinidin-hexoside, cyanidin-3-rutinoside, paeoniflorin 3-glucoside, and paeoniflorin 3-rutinoside [64]. These compounds are relatively rare in ordinary rice varieties, and they endow black rice with unique color and nutritional value [65]. Studies indicate that anthocyanins can chelate with metal elements such as calcium, magnesium, iron, and aluminum. This property contributes to the higher concentrations of calcium, iron, manganese, selenium, and zinc observed in purple and black rice compared to ordinary white rice [66]. Anthocyanins can promote the regeneration of rhodopsin in retinal cells, which helps protect eye health, prevent and improve eye problems such as vision loss and macular degeneration. It can also inhibit the production and release of inflammatory mediators, reduce inflammatory reaction, promote the proliferation of intestinal probiotics, and play a certain auxiliary role in alleviating inflammatory diseases such as enteritis, preventing cardiovascular diseases, diabetes and other important physiological functions [67,68]. A comparative study by Japanese scholars analyzed functional components such as gamma-oryzanol, anthocyanins, flavane-3-ol, chlorogenic acid, flavonoids, and flavonols in black, red, and white rice. The findings revealed that while black rice had the highest anthocyanin content (followed by red rice), red rice was richest in flavan-3-ol, with black rice ranking second [69]. Thailand focuses on the exploration of red rice resources and has found that it is rich in anthocyanins and GABA [70]. Therefore, through genetic engineering, utilizing endosperm-specific promoters to drive key genes for anthocyanin synthesis in rice endosperm is of great significance for the development of colored functional rice in the later stage.

### 3.4. Metabolic Synthesis of Functional Rice

Functional rice integrates multiple active ingredients through metabolic synthesis pathways, forming unique nutritional and functional advantages. Its metabolic network is based on primary metabolism (such as the synthesis of sugars, fats, and proteins), and enriches flavonoids through secondary metabolic pathways including GABA and the synthesis of active substances such as amino acids. Conduct in-depth research on the unique or enriched active substances in functional rice, such as flavonoid biosynthesis pathways, GABA synthesis pathways, etc. [71]. This study investigated GABA synthesis and metabolism mechanisms in giant embryo rice using the variety “Giant Embryo Black Nuo 1”, with the goal of identifying optimal germination conditions to enhance GABA content. Transcriptome and metabolomics will be used to analyze the GABA synthesis and metabolism processes in the grain filling and germination stages of Giant Embryo Black Nuo 1. This study provides a theoretical foundation for the future development and application of GABA-enriched products [62]. By using targeted metabolomics technology, key substances in metabolic pathways are identified, metabolite levels are detected, and their synthesis and accumulation mechanisms are revealed. Functional validation of candidate genes is conducted through the use of gene editing techniques such as CRISPR/Cas9 or transgenic technology. By knocking out or overexpressing specific genes and observing their effects on functional rice metabolism and synthesis, key genes can be identified [72,73]. We can also study the effects of exogenous substances (such as fertilizers, pesticides, environmental factors, etc.) on the metabolic synthesis of functional rice through exogenous regulation methods. By adding or changing environmental conditions externally, observe their effects on metabolic pathways, key gene expression, and accumulation of active substances, and provide a theoretical basis for optimizing cultivation measures.

## 4. The Nutritional Quality and Health Benefits of Colored Rice

### 4.1. Nutritional Evaluation Model and Indicator System

#### 4.1.1. Basic Nutritional Evaluation Indicators

As a staple food, colored rice possesses unique nutritional advantages over white rice due to its core nutritional supply capacity. Its nutritional evaluation system has established a multi-index framework encompassing basic nutrition and functional components, and various scientific evaluation models have been developed (Table 2). In terms of basic nutritional evaluation, the core indicators primarily include five key parameters: carbohydrates, centered around the fine structure of starch, encompassing amylose content, amylopectin chain length distribution, and resistant starch content. As a functional carbohydrate involved in blood glucose regulation, resistant starch is found at significantly higher levels in black rice and brown rice compared to white rice [74]. Protein evaluation considers both the overall content and the completeness of amino acid composition, especially the proportion of essential amino acids such as lysine and tryptophan. Gluten in black rice, due to its high lysine content and digestibility, serves as a high-quality protein source [75]. Lipid quality assessment emphasizes the proportion of unsaturated fatty acids, which in black rice—including oleic and linoleic acid—constitute more than 70% of total lipids. Black rice also contains abundant functional lipids such as γ-oryzanol and β-sitosterol [76]. Mineral and vitamin indicators cover macronutrients such as calcium and magnesium, as well as micronutrients such as iron and zinc. Black rice exhibits significant enrichment characteristics in magnesium, phosphorus, zinc content, and vitamin E and B vitamins, which are core indicators for evaluating its micronutrient supply capacity [75]. In terms of dietary fiber, the dietary fiber content of red rice can reach 4.4 g/100 g, with soluble fiber aiding in weight management and insoluble fiber preventing constipation. The overall content is 5–8 times that of white rice [77]. These basic nutritional indicators collectively constitute the nutritional support of colored rice as a staple food and are also important characteristics that distinguish it from white rice.

Functional components are the core characteristic indicators for the nutritional evaluation of colored rice and an important manifestation of its nutritional advantages. They primarily include flavonoids, phenolic acids, γ-aminobutyric acid (GABA), and so on. Among them, anthocyanins are characteristic components of black rice and purple rice. Various types such as cyanidin 3-glucoside and malvidin 3-galactoside have been isolated and identified from pigmented rice [78]. Cyanidin-3-glucoside (C3G) and peonidin-3-glucoside (P3G) are the main evaluation markers, with a total content ranging from 27.2 to 5045.6 μg/g, and the proportion of C3G can reach 88–100%. Procyanidins are the main functional components of red rice and brown rice, with a maximum content of up to 3060.6 μg/g. Their polymerization degree is positively correlated with antioxidant activity [79,80]. Phenolic acids, including water-soluble and bound forms, are primarily evaluated based on ferulic, p-coumaric, and vanillic acid content. Black rice exhibits significantly higher levels of these phenolic acids than white rice. Additionally, across different origins, the minimum polyphenol content in black rice far exceeds that found in red and purple rice varieties [81]. In addition, GABA, as a non-protein amino acid functional component, can reach a content of 4924.82 ng/mg in germinated black rice and giant embryo black rice, making it an important indicator for evaluating its neuroregulatory-related nutritional functions [75].

#### 4.1.2. Functional Ingredient Evaluation Criteria

Based on the aforementioned basic nutritional and functional component indicators, existing research has established a relatively unified core framework for the nutritional evaluation index system of colored rice, which mainly encompasses three major categories of core indicators: first, basic nutritional component indicators such as protein and starch, which serve as the core basis for measuring basic nutritional value [82,83]; second, the mineral supplementation potential of colored rice is reflected in its content of 8–13 elements, including iron and zinc. Third, its antioxidant and health-promoting properties are closely related to the presence of 9 or more active substances, such as polyphenols, flavonoids, and procyanidins—all of which are found in significantly higher concentrations in black rice compared to red and white rice varieties [82]. On this basis, some studies have further expanded the indicator dimensions by incorporating functional activity indicators such as free radical scavenging activity into the system. For example, Rebeira et al. used free radical scavenging activity, along with indicators such as crude protein and total dietary fiber, as the core evaluation basis when evaluating rice breeding lines, making the evaluation more aligned with nutritional and health-care needs [84]. The team from Shanxi University of Technology also identified differential metabolites such as carotenoids and flavonoids through LC-MS/MS targeted metabolomics technology, providing a new direction for the precise optimization of the indicator system [85].

In terms of constructing nutritional evaluation models, multivariate statistical analysis methods remain the mainstream technical approach, among which the integrated application of PCA and cluster analysis is the most prevalent. Qin et al. selected 5 black rice varieties, 4 red rice varieties, and 10 white rice varieties, measured 22 nutritional indicators, and extracted 5 principal components through PCA [82]. Combined with cluster analysis, they clearly distinguished colored rice from white rice. Xu et al. [83] used 3 red rice varieties, 8 black rice varieties, and 8 ordinary brown rice varieties as materials, extracted 6 principal components based on 27 indicators, and similarly achieved effective differentiation between colored rice and ordinary brown rice. Such models simplify the evaluation dimensions by screening core indicators, achieve objective grading of nutritional quality, and can be directly applied to variety screening. With the development of detection technology and algorithms, evaluation models are gradually upgrading towards rapidity and precision. Related research has achieved rapid nutritional characterization through the combination of nuclear magnetic resonance (NMR) and decision tree analysis (DTA) [86]. In addition, Rebeira et al. evaluated the nutritional quality of 9 advanced rice breeding lines and their parents, combining evaluation models with breeding practices, providing a scientific basis for the cultivation of high-quality colored rice varieties [84].

### 4.2. Potential Intervention Mechanisms for Chronic Diseases

**Figure 5:**
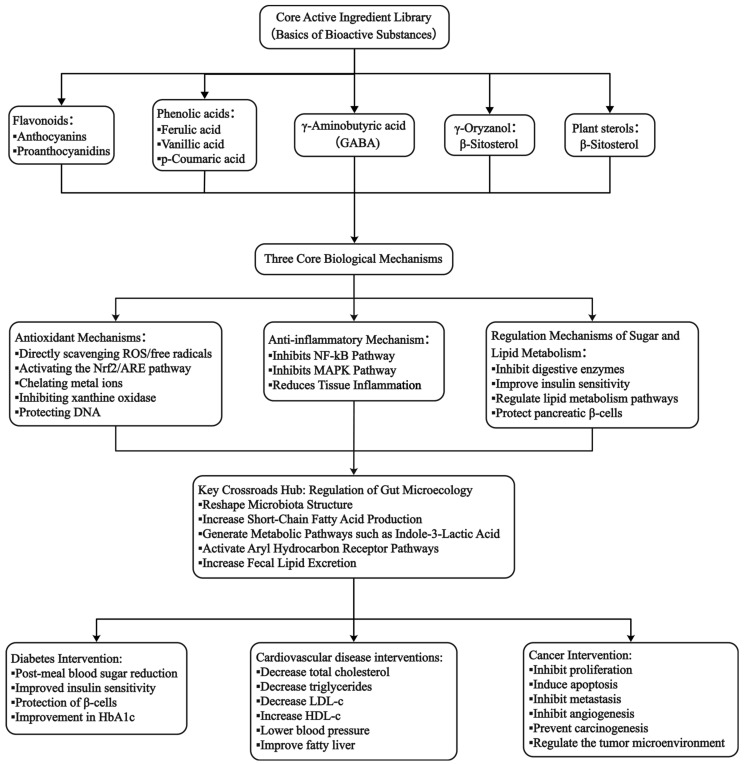
Intervention mechanism diagram of colored rice on chronic diseases.

Rich in bioactive components such as anthocyanins, proanthocyanidins, and phenolic acids, colored rice exerts multi-target intervention effects against chronic diseases—including diabetes, cardiovascular diseases, and cancer—through antioxidant, anti-inflammatory, and glucose-lipid metabolism regulatory mechanisms (Figure 5).

#### 4.2.1. Antioxidant Mechanism

Flavonoids and phenolic acids are the primary sources of antioxidant activity in colored rice. They function by directly scavenging reactive oxygen species (ROS), enhancing the antioxidant defense system, and acting as free radical acceptors to block chain reactions. The appropriate introduction of exogenous antioxidants can alleviate or improve diseases caused by free radicals [87]. Black rice anthocyanins, mainly C3G, exhibit significantly stronger antioxidant activity than white rice in vitro, with DPPH and ABTS radical scavenging rates reaching 75–90% and a ferric reducing antioxidant potential (FRAP) of 35.14–41.25% [77].

Red Rice noodles and white Rice noodles were added to the feed of ordinary rats. By comparing the blood lipids of the two animal groups, they showed that red rice had the effect of increasing plasma HDL and enhancing the body’s antioxidant capacity [88]. Shen et al. [89] measured the total antioxidant capacity of white rice, black rice, and red rice using the ABTS assay. Antioxidant capacity, as measured by ABTS assay, increased progressively from white rice (0.196 mmol/L TEAC) to red rice (1.705 mmol/L TEAC) to black rice (4.484 mmol/L TEAC), with black rice exhibiting values approximately three-fold higher than white rice. Piazza et al. [90] reported that black rice exhibits potential anti-inflammatory and antioxidant effects at the intestinal level. Similarly, Wu et al. [91] used quantitative real-time PCR to measure inflammatory cytokine expression in liver tissue and concluded that black rice pigment alleviates liver inflammation induced by a high-fat diet. Min et al. [92] conducted in vitro and in vivo studies on anthocyanin-3-glycosides and their metabolites in black rice, and found that anthocyanin-3-glycosides exhibit strong anti-inflammatory effects by regulating the activation of NF-κB and MAPK. Anthocyanins enhance the body’s antioxidant defense through dual mechanisms: both by chelating metal ions and inhibiting oxidases (such as xanthine oxidase) to block ROS generation, and by activating the Nrf2 pathway to upregulate antioxidant genes such as HO-1 and NQO1 [91].

#### 4.2.2. Blood Sugar-Lowering Mechanism

As a functional cereal abundant in polyphenols, anthocyanins, and γ-oryzanol, colored rice exhibits hypoglycemic effects supported by in vitro, animal, and clinical studies, operating through a multi-pathway synergistic mechanism. Studies indicate that colored rice primarily regulates blood glucose levels through three core pathways: suppression of carbohydrate digestion, enhancement of insulin sensitivity, and protection of pancreatic β-cells [93]. In terms of inhibiting carbohydrate digestion and absorption, in vitro experiments have confirmed that extracts from black rice and red rice can inhibit α-amylase and α-glucosidase by up to 30–50%. The key active ingredient, cyanidin-3-glucoside (C3G), and procyanidins can significantly reduce postprandial blood glucose spikes and decrease glucose absorption efficiency in the intestine by delaying the hydrolysis of starch into glucose [94,95]. Due to the enrichment of active ingredients, germinated colored rice exhibits superior inhibitory activity against these two key metabolic enzymes, further enhancing its potential for sugar control. The hypoglycemic effect of active ingredients such as anthocyanins in colored rice relies on their stability and bioavailability during gastrointestinal digestion. Anuyahong et al. [96] incorporated Thai purple rice (Riceberry rice), which is rich in anthocyanins, into a yogurt matrix. This not only effectively retained the antioxidant activity of anthocyanins in purple rice but also, in an in vitro gastrointestinal digestion simulation system, the yogurt matrix could effectively delay the degradation rate of anthocyanins, enhancing their bioavailability in the intestine. This provides a food application-level guarantee for the full exertion of anthocyanins in colored rice in terms of enzyme inhibition, antioxidant activity, and other sugar control-related functions, and also completes the action chain of active ingredients from their existing forms to physiological functions.

In terms of improving insulin sensitivity, animal experiments have shown that total flavonoids from red rice can enhance insulin sensitivity in type 2 diabetes mellitus (T2DM) rats by regulating the PI3K/AKT signaling pathway, while simultaneously modulating the gut microbiota structure (such as increasing the abundance of beneficial bacteria) and short-chain fatty acid metabolism, resulting in a synergistic effect on blood glucose regulation [94]. Black rice anthocyanins can improve insulin resistance by activating the expression of genes related to the insulin signaling pathway [5]. The protective effect on pancreatic β cells is primarily achieved through anti-oxidative stress. Polyphenols in black rice extract increase hepatic SOD and GSH-Px activity while decreasing MDA content, thereby protecting pancreatic β-cells from oxidative stress damage and maintaining their insulin secretion function [97].

Human clinical trials have further verified the clinical application value of colored rice: a 3-month intervention with germinated black rice in patients with type 2 diabetes mellitus complicated with dyslipidemia significantly improved glucose and lipid metabolism indicators, while regulating intestinal flora balance; after acute intake of 100 g of black rice by healthy individuals, plasma total phenolic and flavonoid contents significantly increased, and DPPH and ABTS antioxidant activities increased by 70.5% and 62.3%, respectively, with the effect lasting for 180 min, providing antioxidant support for maintaining glycemic homeostasis [98]. Therefore, colored rice exerts hypoglycemic effects through multi-mechanistic synergistic action, and the specificity of its active components, variety differences, and digestive stability in food matrices provide important theoretical basis and practical directions for the development of precision glycemic control foods [96].

#### 4.2.3. Lipid-Lowering and Cardiovascular Protection Mechanisms

The lipid-lowering and cardiovascular protective effects of colored rice primarily rely on the direct regulation of lipid metabolism by its abundant bioactive components. Numerous studies have confirmed that anthocyanins in colored rice are one of the core active substances. In obese mouse models, black rice anthocyanins can significantly alleviate hyperlipidemia, hepatic steatosis, and other issues by regulating lipid metabolism-related pathways, while also improving insulin resistance. This process is closely related to the regulation of gut microbiota, further confirming its intervention value in lipid metabolism disorders [99]. In hyperlipidemic rat models, whole-grain colored rice anthocyanins can directly act on key indicators of lipid metabolism, reducing visceral fat weight, total cholesterol, triglycerides, and low-density lipoprotein (LDL) levels in rats. At the same time, they regulate the gene cascade of peroxisome proliferator-activated receptor γ (PPARγ), which is a core target for lipid metabolism regulation, and its expression regulation directly affects lipid balance [100]. Furthermore, in vitro experiments have confirmed that black rice extracts can inhibit cholesterol absorption through multiple pathways, including inhibiting pancreatic lipase activity, reducing micellar cholesterol solubility, and decreasing cholesterol uptake by intestinal cells. Anthocyanins are the main contributors to these effects, providing direct mechanistic support for their lipid-lowering action in vivo [99].

The intervention effect of colored rice is also reflected in its comprehensive regulation of cardiovascular and metabolic risk factors, exhibiting characteristics of dose-dependence and persistence. Studies have shown that acute intake of colored rice can rapidly enhance plasma antioxidant activity, while simultaneously reducing postprandial blood glucose and insulin levels, and mitigating factors inducing lipid metabolism disorders; whereas long-term consumption can significantly lower fasting blood glucose, body weight, and diastolic blood pressure, indicating that colored rice has a dual effect of short-term relief and long-term improvement on cardiovascular health [101]. Research on specific populations has further expanded its application value. In an ovariectomized rat model, consumption of germinated colored rice can significantly improve lipid profiles, reduce total cholesterol levels, increase high-density lipoprotein cholesterol (HDL) levels, and simultaneously increase fecal lipid excretion, reduce liver lipid accumulation, and effectively lower the risk of dyslipidemia [102]. Intervention experiments on hyperlipidemic mice using purple wheat paste have shown that different doses of purple wheat paste can reverse weight gain in mice, increase serum HDL levels, reduce total cholesterol, triglyceride, and LDL levels, and simultaneously alleviate weight loss and blood glucose elevation in type 2 diabetic mice, indicating that the bioactive components of these colored cereals have a universal intervention effect on dyslipidemia related to metabolic disorders [103].

Therefore, colored rice achieves lipid-lowering and cardiovascular protective effects through its rich bioactive components, such as anthocyanins, by regulating lipid metabolism pathways, modulating gut microbiota, inhibiting cholesterol absorption, and comprehensively improving cardiovascular metabolic risk factors [104].

#### 4.2.4. Antitumor Mechanism

The components such as anthocyanins, procyanidins, and phytosterols in colored rice exert anti-tumor effects by inhibiting tumor cell proliferation, inducing apoptosis, and blocking angiogenesis. Anthocyanins, as the most extensively studied anti-tumor active components in colored rice, exert anticancer effects through multiple pathways. Liu Yaoguang’s team at South China Agricultural University constructed purple rice with high anthocyanin content through genetic modification technology, confirming that rice with high anthocyanin content can significantly reduce cancer risk and clarifying that anthocyanins are the core material basis for the anti-tumor effect of colored rice. Specifically, anthocyanins can achieve anticancer effects through antioxidation, anti-inflammation, and regulation of the apoptosis pathway. For example, anthocyanins in purple rice bran extracts can reduce cellular inflammatory responses and inhibit abnormal cell proliferation, thereby preventing diethylnitrosamine-induced liver cancer in rats. Cyanidin-3-glucoside in Thai black rice bran can block the epithelial–mesenchymal transition process of human prostate cancer cells by inhibiting the expression of cytoskeleton-related proteins, reducing the invasion and migration abilities of cancer cells, and its effect relies on the regulation of Snail/E-cadherin expression by the Smad signaling pathway [105]. Black rice anthocyanins significantly inhibit ErbB2-positive breast cancer cells. In vivo studies show that oral administration reduces tumor growth and lung metastasis in nude mice, accompanied by concentration-dependent suppression of cancer cell migration and adhesion, as well as downregulation of urokinase-type plasminogen activator activity [106].

Except for directly acting on cancer cells, colored rice can also exert an anti-tumor effect indirectly by regulating intestinal microecological balance. This mechanism has been fully validated in colorectal cancer prevention and treatment research. A black rice diet can significantly reduce the size and number of intestinal tumors in colorectal cancer model mice (ApcMin/+ and AOM/DSS), extending the lifespan of the mice. Mechanistic studies showed that black rice consumption reshapes gut microbiota—enriching Bacteroides singuliformis and Lactobacillus—while increasing intestinal metabolites such as indole-3-lactic acid and indole. These metabolites inhibit colorectal cancer cell proliferation and tumorigenesis by specifically activating the aryl hydrocarbon receptor (AHR) pathway. Fecal transplantation experiments further confirmed that the intestinal microecological changes induced by a black rice diet are the key mediating factors for its anti-tumor effect [107]. Anthocyanins can exert an indirect anti-tumor effect by regulating the functions of immune cells in the tumor microenvironment, particularly by modulating the polarization of tumor-associated macrophages (Tumor-Associated Macrophages, TAMs) and enhancing the activity of natural killer (Natural Killer, NK) cells. In terms of macrophage polarization regulation, anthocyanins can promote the polarization of TAMs from the tumor-promoting M2 phenotype to the anti-tumor M1 phenotype. In terms of NK cell activity, studies have shown that the expression of the activation receptor NKG2D on the surface of NK cells, as well as the secretion of perforin and granzyme B, are the key factors for NK cells to exert their tumor-killing function [105].

In addition, other active proteins in colored rice also possess potential anti-tumor-related functions. Research on peroxidase OsPrx3 in red rice has shown that this protein exhibits significant antioxidant activity, effectively scavenging hydroxyl radicals and inhibiting DNA oxidative damage, which is one of the important triggers of cell carcinogenesis. In vitro experiments have demonstrated that OsPrx3 exhibits a clear dose-effect relationship in inhibiting oxidative damage within a concentration range of 0.06 to 0.18 µg/µL, with cysteine at position 51 being the key site for activity [108]. Model experiments using Caenorhabditis elegans have confirmed that ingesting OsPrx3 protein can significantly enhance the body’s ability to resist oxidative stress and reduce the level of reactive oxygen species in the body. Given the strong association between oxidative stress imbalance and tumorigenesis, this protein shows promise for cancer prevention [109].

### 4.3. Population Dietary Intervention and Epidemiological Evidence

As a specialty grain, colored rice has become a hot topic in nutritional epidemiology and dietary intervention research due to its application in human diets and health benefits. Yang et al. [110]. compared the nutritional components of whole grain black rice with whole grain white rice (WGWR), milled black rice (MBR), and milled white rice (MWR). They found that whole grain black rice significantly outperforms other types of rice in terms of nutritional density and bioactive substance content. Its nutrient density unit (NDU) value is as high as 1.11, much higher than the 0.44 of milled white rice. Rich in both basic nutrients (dietary fiber, minerals, vitamins) and bioactive compounds (anthocyanins, procyanidins), colored rice represents a paradigm shift from calorie provision to enhanced nutritional functionality, thereby establishing a robust foundation for future human dietary intervention research [110].

This nutritional omics analysis reveals the multiple nutritional advantages of whole-grain black rice: at the macronutrient level, its protein content is 39% higher than that of polished rice, dietary fiber content is 203% higher, and fat content is 284% higher; at the micronutrient level, the content of minerals such as calcium, phosphorus, and potassium increases by 137–1158%, vitamin B group content increases by 192–541%, and vitamin E content reaches 1467.3 μg/100 g. Moreover, these nutrients are prone to significant loss during the polishing process. The nutritional advantages of whole-grain black rice are also reflected in resource utilization efficiency. Using the whole-grain form can increase the edible portion of black rice by 44.2% compared to polished rice, which not only enhances nutritional value but also ensures food security, providing socioeconomic support for its large-scale dietary promotion. Meanwhile, the research team has cultivated a new variety, “Huamoxiang No. 3”, which optimizes the cooking quality and taste while retaining the nutritional advantages of whole-grain black rice. This variety breaks through the palatability bottleneck in the promotion of colored rice, providing variety support for the implementation of population dietary intervention [110].

In terms of regulating cardiometabolic risk factors, systematic reviews have identified the positive effects of consuming colored rice. A systematic review encompassing 17 randomized controlled trials found that acute intake of colored rice significantly enhances plasma antioxidant activity, while effectively reducing postprandial blood glucose and insulin levels. Long-term regular consumption of colored rice is associated with decreased fasting blood glucose, weight loss, and reduced diastolic blood pressure, providing important theoretical evidence for the application of colored rice in the prevention of cardiometabolic diseases [104]. Targeted intervention studies for specific populations further validate this conclusion. For example, a crossover clinical trial revealed that consuming different colored rice varieties significantly enhances antioxidant activity in obese individuals. Specifically, Purple rice consumption led to a 40.3% increase in total antioxidant activity after one hour, compared to 29.5% at 30 min and 21.2% at one hour for Yunlu29 red rice. Additionally, it has a regulatory effect on inflammatory biomarkers in the body, suggesting that colored rice can improve the metabolic status of obese individuals through both antioxidant and anti-inflammatory pathways [111].

In the dietary management of patients with chronic diseases, the intervention effect of colored rice has also been fully confirmed. Intervention studies targeting individuals with type 2 diabetes mellitus (T2DM) complicated by dyslipidemia have shown that compared to regular white rice, consumption of germinated brown rice and germinated black rice as dietary interventions significantly modulates patients’ blood glucose and lipid metabolism markers. This mechanism likely operates through multi-pathway synergy: regulating adipokines and lipid-metabolizing enzymes, while also enriching gut microbiota diversity and short-chain fatty acid-producing bacteria to boost SCFA levels. This provides new ideas and practical directions for optimizing dietary intervention programs for individuals with T2DM complicated by dyslipidemia [112]. The health benefits of colored rice have been extended to the field of women’s health. Tu et al. [113] revealed the intervention potential of a whole-grain black rice diet for ovarian aging. Ovarian aging is a key biological process affecting women’s fertility and overall health, accompanied by reduced follicular reserve, hormonal imbalance, and deterioration of the ovarian microenvironment. Especially in the context of delayed childbearing age, it has become a public health issue that requires urgent attention. Safe and long-term dietary strategies are more clinically translatable than drug and hormone interventions. Through mouse experiments, the study confirmed that a whole-grain black rice diet can reshape the single-cell transcriptome profile of age-related ovarian decline, improve the ovarian microenvironment at the molecular level, and regulate pathways related to follicular development, providing a novel dietary intervention approach for maintaining female ovarian function. Its efficacy also stems from the rich bioactive compounds and nutrient reserves in whole-grain black rice [113]. Furthermore, the potential of colored rice in the prevention and intervention of neurodegenerative diseases has also been revealed in recent research. The “Preventive Treatment of Diseases” team at Huazhong Agricultural University found that α-linolenic acid (ALA) and 11,14-eicosadienoic acid (EDA), abundant in colored rice (black rice), can inhibit the amyloid pathology of Alzheimer’s disease by allosteric activation of the cell-specific GPR120 receptor. The study further elucidated that EDA can enhance the binding affinity of ALA with GPR120, promoting the phagocytosis and clearance of Aβ plaques by activating the GPR120-Gαi-mTORC1 signaling pathway in Aβ plaque-associated macrophages and activated microglia. This, in turn, improves the cognitive function and prolongs the lifespan of model animals. This mechanism provides novel molecular biological evidence for the application of colored rice in the “preventive treatment” of neurodegenerative diseases [114].

## 5. The Industrial Application Prospects and Challenges of Colored Rice

### 5.1. Innovation in Breeding and Cultivation Technologies

**Figure 6:**
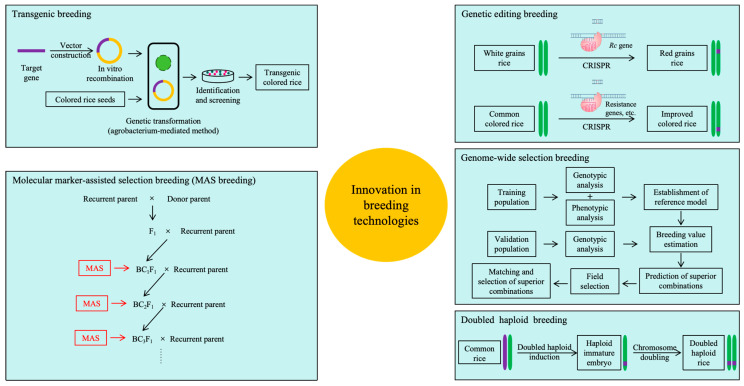
Innovation in breeding technologies for colored rice.

As an important functional cereal resource, the primary step in the industrial development of colored rice is variety breeding and cultivation technology innovation. Although colored rice possesses unique advantages due to its rich content of nutrients such as anthocyanins, traditional varieties generally suffer from issues such as long growth period, low yield, and weak stress resistance, which restrict industrial development [44]. In recent years, diverse innovations in breeding technology and optimization of cultivation methods have provided core support for the improvement and quality enhancement of colored rice varieties (Figure 6; Table 3).

Due to its long cycles, low efficiency, and vulnerability to environmental factors, traditional breeding struggles to precisely improve complex quantitative traits [115,116]. With the development of bioscience and technology, innovations have been made in colored rice breeding technology, including transgenic technology, molecular marker-assisted selection, gene editing technology, genome-wide selection technology, and double haploid technology [117]. This provides key support for breaking the bottleneck of colored rice industrialization and cultivating high-quality new varieties. In transgenic breeding, Zhu et al. designed the de novo biosynthesis of anthocyanins and astaxanthin in rice endosperm, assembled relevant synthetic genes using the high-efficiency multi-gene stacking system TGSII, and developed “Purple Crystal Rice” and “Red Crystal Rice” through transgenic technology [118]. The team also created “Ruby Rice” with endosperm rich in betacyanin by co-expressing the feedback-insensitive tyrosine dehydrogenase (ADHα) and the RUBY system [20]. To break through the efficiency bottleneck of traditional methods, molecular marker-assisted selection has become a basic tool for precision breeding, with its core technology being the development of functional markers closely linked to target traits (such as taste value and pigment) [119]. In terms of gene editing technology, Zhu et al. (2019) used CRISPR-Cas9 technology to edit the *Rc* gene, successfully transforming a white cultivated rice variety into a red rice variety [120]. Sedeek et al. used CRISPR-Cas9 technology to precisely knock out three flowering inhibitory genes, Hd2, Hd4, and Hd5, improving the flowering time and plant height of the black rice variety Cempo Ireng [44]. In addition, Lu et al. believe that rapidly accumulating excellent genes regulating agronomic traits in the context of colored rice through whole-genome selection breeding will be an effective means to cultivate new varieties of colored rice with balanced nutrition and yield traits [39]. Liu et al. successfully developed the rice haploid induction line Hi285 with a haploid induction efficiency of 12.4% using double haploid technology [121]. However, there are currently no reports on the application of double haploid technology in colored rice. Based on the current status of rice breeding, modern colored rice breeding needs to focus on the deep integration of traditional breeding methods and modern biotechnology while applying new breeding technologies [122,123].

In recent years, significant progress has been made in research on cultivation technology innovation, focusing on the synergistic improvement of yield and nutritional quality in colored rice. In terms of light and temperature regulation, studies have shown that red rice is suitable for early sowing to utilize higher light and temperature for anthocyanin accumulation, while black rice, with stronger shade tolerance, is suitable for planting in seasons or regions with lower light and temperature to enhance rice quality [124]. In water management, flooding disrupts plant metabolism and significantly inhibits the synthesis of amino acids in purple rice grains [125]. Another study indicates that increasing irrigation tends to enhance colored rice yield but reduces bioactive substance accumulation [126]. In nitrogen fertilizer management, with the increase in nitrogen application rate, the yield of colored rice improves, and the contents of most amino acids, anthocyanins, flavonoids, and total phenolic compounds all rise [126,127]. Meanwhile, a combined application mode of 30% inorganic nitrogen fertilizer replaced by plant-based organic fertilizer can synergistically enhance the yield and anthocyanin content of colored rice, while improving soil physicochemical properties [128]. In the management of special functional fertilizers, spraying 400 g/ha zinc fertilizer on the leaves during the heading stage can increase the zinc content in polished rice of some colored rice varieties beyond the zinc-rich standard of 45 mg/kg [129]. Spraying 10 mg/L exogenous selenium can simultaneously enhance the nutritional components, bioactive substances, and selenium-rich level of purple rice grains [33]. Application of iron fertilizer via foliar spraying significantly boosts the anthocyanin content of black rice and the proanthocyanidin content of red rice, as well as the total flavonoid levels and antioxidant capacity of both grain types [35]. Spraying amino acids during the heading and grain-filling stages can effectively enhance the nutritional quality and yield of purple rice grains [130]. In terms of plant growth regulators, studies have found that 1.25 μmol/L gibberellin and abscisic acid significantly promote the elongation growth of hypocotyls in etiolated seedlings of black rice, with gibberellin showing a stronger effect than abscisic acid [131]. The rotation of colored rice with green manure has further expanded the green cultivation path. The in situ application of green manures such as soybeans and Buxus chinensis can increase yields by 12.7% to 29.0% and enhance anthocyanin content by 7.2% to 24.2% [132]. However, the current colored rice industry still lacks a standardized planting technology system. Future development requires integrating a variety of characteristics with regional ecological factors to optimize cultivation parameters and drive high-quality, large-scale industrial growth.

**Table 3:** Research progress and effects of innovation in pigmented rice cultivation technologies.

Technology Category	Specific Measures/Findings	Main Effects	References
Light and temperature regulation	Red rice is suitable for higher light and temperature conditions	Promotes the accumulation of anthocyanins and flavonoids	[124]
	Black rice is suitable for lower light and temperature conditions	Promotes the accumulation of anthocyanins and flavonoids	
Water management	Flooding	Inhibits the synthesis of grain amino acids	[125]
	Excessive irrigation	Inhibits the accumulation of bioactive substances	[126]
Nitrogen fertilizer management	Increasing nitrogen application rate	Improves yield and increases the contents of most amino acids, anthocyanins, flavonoids, and total phenols	[126,127]
	Organic-inorganic combined application (30% inorganic nitrogen → 30% organic fertilizer)	Synergistically improves yield and anthocyanin content, and improves soil physical and chemical properties	[128]
Special functional fertilizers	Foliar application of zinc fertilizer at heading stage (400 g/ha)	The zinc content of polished rice of some varieties exceeds the zinc-rich standard (45 mg/kg)	[129]
	Spraying exogenous selenium (10 mg/L)	Simultaneously improves the nutritional components, active substances, and selenium-rich level of purple rice	[33]
	Spraying iron fertilizer	Significantly improves the active substances and antioxidant capacity of black rice and red rice	[35]
	Spraying amino acids at heading and filling stages	Effectively improves the nutritional quality and yield of purple rice grains	[130]
Plant growth regulators	Application of gibberellin and abscisic acid (1.25 μmol/L)	Significantly promotes the elongation of the mesocotyl of etiolated black rice seedlings, with gibberellin having a better effect	[131]
Ecological rotation mode	Colored rice-green manure rotation	Expands the green cultivation path and improves yield and anthocyanin content	[132]

### 5.2. Processing and Storage Technology

#### 5.2.1. Processing Technology

**Figure 7:**
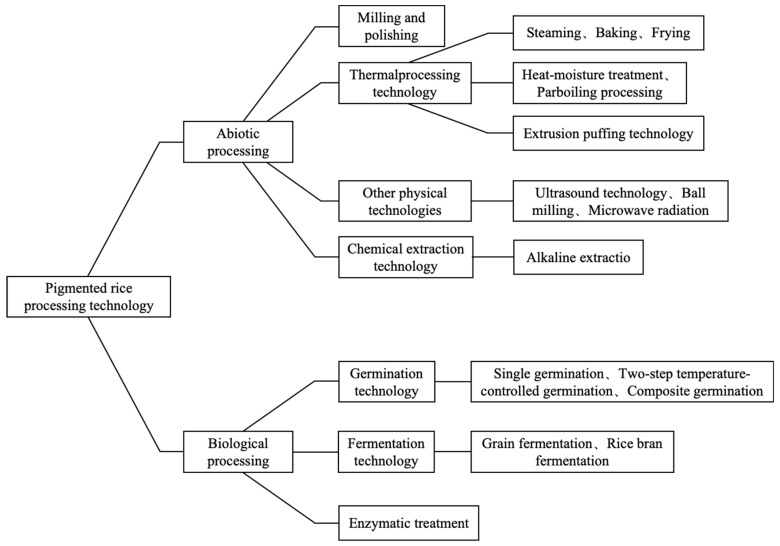
Classification of colored rice processing techniques.

The processing technology of colored rice directly affects its nutrient retention, sensory quality, and processing applicability. Compared to ordinary white rice, colored rice is rich in anthocyanins, phenolic compounds, and other active substances, as well as unique nutrients. Different processing methods significantly affect the nutritional components and physiologically active substances of colored rice. Existing studies have concentrated on two principal processing approaches: abiotic treatments and biotic treatments. Choosing appropriate processing technology can significantly reduce the loss of nutrients (Figure 7) [133].

Milling and polishing are essential steps in the processing of colored rice into polished rice, and their processing intensity directly determines the degree of nutrient retention [134]. Since the nutritional components are mainly concentrated in the bran layer and the outer layer, the milling process significantly reduces the levels of active substances such as phenols, flavonoids, and anthocyanins, as well as the antioxidant capacity in colored rice [135]. At the same time, milling significantly improves the cooking quality of colored rice and enhances sensory evaluation [136]. Therefore, moderate milling (with a milling degree controlled at 3% to 5%) is key to balancing nutrient retention and edible quality in colored rice.

Cooking methods such as steaming and baking are the main ways for colored rice to be consumed directly or processed into products. After steaming, the content of total phenols (TP), procyanidins (OPC), flavonoids, total antioxidant activity, and some nutrient elements in colored rice significantly decreases [137]. Microwave steaming significantly enhances TP, total anthocyanin content (TAC), DPPH, and FRAP of colored rice compared to steam steaming [138]. Meanwhile, microwave treatment significantly reduces the rapidly digestible starch (RDS) content of black rice and increases the slowly digestible starch (SDS) and resistant starch (RS) components [139]. Baking significantly increases the TPC and radical scavenging activity of colored rice. At the same time, the content of flavonoids decreases, with a significant reduction in anthocyanin content [140]. Frying enhances the α-glucosidase activity, amylose, and resistant starch content of colored rice [141]. Heat-moisture treatment leads to a decrease in the solubility and RDS of purple rice noodles, while increasing the RS and SDS values. Simultaneously, moist heat treatment reduces the total phenol content, total anthocyanin content, and the scavenging activity of DPPH and ABTS radicals in purple rice noodles [142]. Parboiling is a special steaming pretreatment technique that includes soaking, steaming, drying, grinding, and polishing [143]. Parboiling treatment leads to a significant decline in the antioxidant capacity of black rice, attributed to reductions in anthocyanins, total phenols, procyanidins, and total flavonoids, according to research findings [144].

Extrusion-expansion technology is an innovative technique that integrates mixing, curing, and expansion molding. It boasts advantages such as simple operation, high raw material utilization, minimal nutrient loss, resistance to product reversion, and good storage stability [145]. Extrusion-expansion significantly reduces the content of fat, starch, total phenols, and anthocyanins in black rice, with no significant change in protein content. It notably enhances the solubility characteristics and significantly reduces the antioxidant capacities of DPPH, ABTS, and FRAP [146]. High temperature and high shear force cause the degradation and decarboxylation of phenolic acids, which may be the reason for the decrease in phenolic acid content in total phenols [147]. Furthermore, extrusion-expansion can also increase the diversity of volatile compounds in black rice/purple rice [148].

In addition to the aforementioned non-biotechnological methods, there are also physical techniques such as ultrasonic and ball milling, as well as chemical techniques such as alkaline extraction. Ultrasonic technology can assist in enhancing the extraction efficiency of antioxidant components from colored rice bran [149]. Ultrasonic and ball milling techniques are used to alter the physical properties of colored rice starch [150]. Microwave radiation can increase the content of bioactive compounds that inhibit α-amylase, thereby reducing starch digestibility [151]. Alkaline extraction is a core chemical technique for extracting components such as protein and polyphenols from colored rice [152,153]. Cold Plasma (CP) technology generates highly reactive particles by ionizing gases, enabling surface modification and sterilization of materials. It is a green non-thermal technology that causes minimal damage to heat-sensitive components such as anthocyanins in colored rice. High Hydrostatic Pressure (HHP) is a non-thermal technology that applies static water pressure at room temperature or low temperatures to achieve sterilization and enzyme activation. It also enhances the bioavailability of anthocyanins [154].

Germination, fermentation, and enzymatic treatment are three primary bioprocessing methods. Germination and fermentation enrich active components and improve quality by activating endogenous enzyme activity or utilizing microbial metabolism in colored rice. Germination enhances the nutritional content, GABA, and bioactive substances of purple rice, and softens the bran layer, improving the taste [155]. Traditional rapid germination methods for rice are inefficient, but a two-step temperature-controlled germination process significantly increases the bioactive substance content, antioxidant activity, and enzyme inhibitory capacity of colored rice [156]. Furthermore, germination can be combined with technologies such as ultrasound [157] and zinc fortification [158] to further improve the nutritional and sensory properties of colored rice products. Microbial fermentation treatment significantly enhances the antioxidant activity of colored rice [159], significantly increases total phenolic content [160], and increases free phenolic content [161]. Rice bran from colored rice is often subjected to fermentation treatment, which significantly increases total phenolic content and decreases total flavonoid and anthocyanin content [162]. Enzymatic treatment can be used to extract proteins, polyphenols, and other components from colored rice for the development of novel food ingredients [152,153]. Furthermore, it can be combined with germination technology to significantly improve the total phenolic content and antioxidant activity of colored brown rice [163].

#### 5.2.2. Storage Technology

Colored rice is rich in lipids and bioactive compounds, and during storage, it is prone to quality deterioration such as oxidative rancidity, microbial contamination, insect infestation, and nutrient degradation [164,165]. Therefore, appropriate treatment methods are needed to extend storage time while preserving the nutritional and sensory qualities of the rice. Drying methods, temperature and humidity, modified atmosphere techniques, packaging methods, and pre-storage processing all affect the nutrient content and storage duration of colored rice [166].

Before storage, rice needs to undergo drying treatment. Due to the susceptibility of phenolic compounds such as anthocyanins and phenolic acids in colored rice to thermal degradation, solar drying (≤30 °C) is more effective than hot air drying (40 °C) in reducing the loss of active components such as anthocyanins in black rice [167]. Meanwhile, some scholars suggest that the drying temperature for red rice should be below 60 °C [168]. Temperature has a significant impact on the storage quality of colored rice, and low-temperature storage can delay lipid oxidation and nutrient degradation [169]. The humidity of the storage environment needs to be controlled within an appropriate range. Excessive humidity will accelerate the oxidation of fat in the rice embryo and the degradation of proteins, while insufficient humidity may cause micro-cracks in the grains [170]. Controlled atmosphere storage can minimize the harm caused by chemical pesticides to warehouse keepers, protect the environment and the grain itself from chemical contamination, and also has good insecticidal and mold-inhibiting effects and can delay the aging rate of rice [171]. Compared to vacuum and atmospheric storage environments, nitrogen storage is the optimal condition for maintaining the phenolic compounds in black rice [172]. Furthermore, research has shown that two packaging materials, polyethylene/ethylene-vinyl alcohol copolymer/polyamide/polyethylene and polyamide/polyethylene surface embossing, can effectively delay the quality deterioration of purple rice [173]. Ferreira et al. [174] found that UV-C radiation is an effective method for controlling fungi and mycotoxins in colored rice. Pre-storage processing also affects the quality changes and storage time of stored colored rice. Two processing methods, drum drying and extrusion expansion, can improve the quality deterioration of black rice caused by lipid oxidation during storage, enhancing the storage stability of colored rice [175]. The greater the milling degree, the more severe the rancidity and loss during black rice storage [176].

### 5.3. Product Development and Market Expansion

The development of colored rice products centers around “nutrient retention, functional enhancement, and morphological innovation”, combining different processing technologies to form three major product systems: basic processed products, functional foods, and deep-processed products. These cover multiple sub-sectors such as food grains, snacks, beverages, and health products, meeting the needs of different consumer groups.

The basic processed products, centered around unprocessed colored rice, undergo moderate processing to retain their nutritional components. These primarily include high-quality colored brown rice and polished colored rice, which are the mainstream products in the current colored rice market. Furthermore, by combining colored rice with minor grains such as buckwheat and pearl barley, or refined white rice, products like mixed minor grain rice and formulated rice have been developed, further enriching product forms and meeting consumers’ diverse needs for healthy staple foods [177,178].

Functional food is the core direction of colored rice product development. Leveraging its rich active ingredients, such as anthocyanins and phenolic compounds, foods with antioxidant, hypoglycemic, and immune-enhancing functions are developed, aligning with the current trend of health consumption upgrading. Currently, functional products such as purple rice paste, purple rice noodles [179], purple rice bread [180], and sprouted colored brown rice meal replacement have been developed. For example, purple brown rice instant meal replacement powder prepared using extrusion technology [181]. Sprouting technology is used to increase the GABA content in colored rice, and then processed to produce sprouted black rice instant congee [182]. In addition, colored rice also exhibits unique advantages in the development of gluten-free foods, replacing some cereal raw materials to prepare gluten-free bread, noodles, and other products, meeting the consumption needs of people with special dietary requirements [183,184].

Deep-processed products are made from colored rice as raw material, utilizing techniques such as fermentation and extraction to prepare high-value-added products, significantly enhancing the added value of the colored rice industry. For instance, black rice wine, black rice vinegar, purple rice yogurt, and colored rice lacto beverages have been developed by utilizing microbial fermentation [185,186,187,188]. At the same time, the seasonings such as soy sauce prepared from the fermentation of colored rice not only have unique colors but also are rich in various amino acids, enhancing the flavor and nutritional value of the products [189]. Moreover, Jin et al. believe that the active substances in rice are a new direction for increasing added value and are the focus of future industrial development [190]. For example, black rice anthocyanin is utilized to develop new packaging films [191], black rice extract mouthwash [192], etc.; Use purple rice bran soluble dietary fiber, poly (ethyl epoxy) composite nanofibers, used for embedding alpha tocopherol, enhance its stability and promote a controlled release [193].

## 6. Conclusions and Outlook

As high-value functional cereals, colored rice possesses nutritional and health values that far exceed the basic energy supply function of conventional white rice, realizing a full-value chain transformation from field cultivation to human health regulation. The unique functional advantages of colored rice are attributed to abundant bioactive substances including anthocyanins, proanthocyanidins and GABA, whose synthesis and accumulation are regulated by MYB and bHLH transcription factor networks and modulated by light, temperature, fertilizer and other environmental conditions. Rich in macro and micronutrients, colored rice can exert antioxidant, anti-inflammatory, glycolipid-regulating and gut microbiota-modulating effects, providing scientific support for the homology of medicine and food and promising intervention potential against chronic metabolic diseases. Modern molecular breeding and intelligent processing technologies have effectively broken traditional technical bottlenecks, driving the colored rice industry to form an integrated innovation system covering germplasm improvement, precision cultivation, nutrition-oriented processing and health-driven market, and promoting the strategic transformation of the rice industry from quantity-oriented supply to health-oriented consumption. Nevertheless, the industrialization of colored rice still faces systematic constraints. The narrow genetic basis of germplasm resources and the linkage of unfavorable agronomic traits hinder the synergistic improvement of high yield and high nutritional quality, while the application of advanced gene-editing breeding technologies is restricted by public cognition and regulatory policies. In processing production, the enrichment of functional ingredients in rice bran and embryos conflicts with traditional fine processing modes, and the vulnerability of active substances to heat, light and oxygen causes nutrient loss, limiting the improvement of industrial added value. In addition, high market positioning, insufficient popular science education, and disjointed production–processing–sales links lead to serious product homogenization and low market penetration.

Future research on colored rice requires interdisciplinary systematic innovation to achieve precise nutritional improvement and industrial upgrading. Cutting-edge omics technologies can help clarify the molecular regulatory mechanisms of active substance accumulation and environmental interaction, while the integration of synthetic biology and food engineering supports the innovative breeding of nutrient-efficient rice varieties and the development of novel functional foods with high bioavailability. Large-scale cohort studies and real-world data analysis are also essential to establish accurate dose–effect relationships and population-specific nutritional intervention evidence, forming a complete scientific evidence chain from molecular mechanisms to health benefits. For industrial high-quality development, it is necessary to incorporate functional colored rice industry into national food security and health strategies, optimize germplasm innovation and intelligent design breeding, and establish unified whole-industry-chain standards for cultivation, processing and quality traceability. Meanwhile, strengthening in-depth collaboration among breeding, planting, processing and marketing entities, expanding diversified product layouts in the big health industry, and conducting targeted market popularization and brand building can further enhance the market competitiveness of Chinese colored rice products. In conclusion, colored rice is a key carrier of grain functionalization and nutritional enhancement. Sustained interdisciplinary innovation and whole-industry-chain coordination will facilitate its transformation from a niche specialty industry to a core industry leading healthy staple food consumption, making important contributions to preventing chronic diseases and promoting human health.

## Figures and Tables

**Table 2:** Comparison of core basic nutritional indicators between colored rice and white rice.

Nutrition Category	Key Evaluation Indicators	Colored Rice	White Rice	Conclusions
carbohydrate	Resistant starch	Black rice/brown rice: significantly higher levels of resistant starch.	The content is relatively low.	Resistant starch is associated with blood glucose regulation. Colored rice is superior to white rice in this functional component [74].
Protein	Total Protein; Essential amino acids (lysine, tryptophan)	Black rice: Glutenin, due to its high lysine content and digestibility, serves as an excellent protein source.	The protein quality is relatively low, with lysine being the first limiting amino acid.	Black rice has a higher protein content, a more complete amino acid composition, and a superior biological utilization rate [75].
Lipids	Proportion of unsaturated fatty acids; Functional lipids	Black rice: Unsaturated fatty acids such as oleic acid and linoleic acid account for more than 70% of the total lipids. It is rich in functional components such as γ-oryzanol and β-sitosterol.	Low in fat, primarily saturated fatty acids, and lacking functional lipids.	The lipid quality of colored rice is healthier and contains unique bioactive substances [76].
dietary fiber	Total dietary fiber	Red rice: Its content can reach 4.4 g/100 g. Soluble fiber aids in weight management, while insoluble fiber prevents constipation.	The content is extremely low, and most of the fiber in polished white rice is lost during processing.	The overall dietary fiber content of colored rice is 5–8 times that of white rice, which is one of its core advantages as a healthy staple food [77].
Minerals and vitamins	Trace elements: iron, zinc, etc. Macroelements: magnesium, phosphorus, calcium, etc. Vitamins: Vitamin E, B-complex	Black rice: It exhibits significant enrichment characteristics in magnesium, phosphorus, zinc content, as well as vitamin E and B vitamins.	During the refining process, a significant amount of minerals and vitamins are lost, except for fortified white rice.	Colored rice is a natural and high-quality source of dietary micronutrients, with its bran layer (colored layer) being rich in these components [75].

## Data Availability

Not applicable.
